# A novel molecular diagnostic method for the gut content analysis of *Philaenus* DNA

**DOI:** 10.1038/s41598-021-04422-1

**Published:** 2022-01-11

**Authors:** Isabel Rodrigues, Vítor Ramos, Jacinto Benhadi-Marín, Aránzazu Moreno, Alberto Fereres, José Alberto Pereira, Paula Baptista

**Affiliations:** 1 Centro de Investigação de Montanha (CIMO), Instituto Politécnico de Bragança, Campus de Santa Apolónia, 5300-253 Bragança, Portugal; 2 Departamento de Ingeniería Agrária, Universidad de Léon, Av. Portugal, n° 41, 24071 Léon, Spain; 3 Instituto de Ciencias Agrarias, Consejo Superior de Investigaciones Científicas (ICA-CSIC), C/Serrano 115 dpdo, 28006 Madrid, Spain

**Keywords:** Agroecology, Molecular ecology

## Abstract

*Philaenus spumarius* is a vector of *Xylella fastidiosa*, one of the most dangerous plants pathogenic bacteria worldwide. There is currently no control measure against this pathogen. Thus, the development of vector control strategies, like generalist predators, such as spiders, could be essential to limit the spread of this vector-borne pathogen. In this study, a polymerase chain reaction (PCR)-based approach was developed to principally detect DNA of *P. spumarius* in the spider’s gut. Accordingly, 20 primer pairs, targeting the mitochondrial cytochrome oxidase I (COI) and cytochrome b (*cyt*B) genes, were tested for specificity, sensitivity, and efficiency in detecting *P. spumarius* DNA. Overall, two primer sets, targeting COI gene (COI_Ph71F/COI_Ph941R) and the *cyt*B gene (cytB_Ph85F/cytB_Ph635R), showed the highest specificity and sensitivity, being able to amplify 870 pb and 550 bp fragments, respectively, with *P. spumarius* DNA concentrations 100-fold lower than that of the DNA of non-target species. Among these two primer sets, the cytB_Ph85F/cytB_Ph635R was able to detect *P. spumarius* in the spider *Xysticus acerbus*, reaching 50% detection success 82 h after feeding. The feasibility of this primer set to detect predation of *P. spumarius* by spiders was confirmed in the field, where 20% of the collected spiders presented positive amplifications.

## Introduction

The meadow spittlebug *Philaenus spumarius* (Linnaeus, 1758) (Hemiptera, Aphrophoridae) is the most common and widespread xylem-sap feeder insect in Europe, where it has never been considered as a pest^1^. However, since the first European outbreak of *Xylella fastidiosa* (Xanthomonadales: Xanthomonadaceae) in olive^2^, *P. spumarius* has become a serious threat to European agriculture to its recognized role in the transmission of this pathogen^3^. Up to date, in the south of Italy, thousands of olive trees in an area higher than 23,000 ha, died due to *X. fastidiosa*^4^. *Philaenus spumarius* was identified as the main vector responsible for this severe outbreak^5^. Indeed, although all xylem-sap feeders are considered potential vectors of *X. fastidiosa*^6^, it is proven that species belonging to the genus *Philaenus* have transmission rates higher than other vectors^5^. Therefore, if proper prevention and control measures are not implemented, the continuous dispersion of *X. fastidiosa* over 50 years could cause economic damage higher than 1.9 billion Euros^7^. In this regard, vector control is perceived as the main tool to limit the spread of *X. fastidiosa*^7^. Previous studies showed that vector control measures, based on chemical and physical strategies, can significantly reduce the population of *P. spumarius* and consequently decrease the spread of *X. fastidiosa*^8,9^. However, there is a need for a more sustainable and ecological approach as an alternative to chemical control due to the known ability of pesticides to cause deleterious health and environmental effects.

Generalist predators, such as spiders, can play a significant role in the biological control of *P. spumarius*. Spiders are one of the most abundant and diverse arthropod orders^10^. There are more than 45,000 species of spiders described in the world, and in favourable conditions, they can reach population densities higher than 1000 individuals m^2^^11^. In addition, they are considered one of the most important groups of natural insect enemies worldwide^10^. Spiders are polyphagous and opportunistic predators with different hunting strategies, capable of killing approximately 200 kg/ha of prey per year^12^. Although there are already reports of spiders preying on *P. spumarius*^13,14^, knowledge about its antagonist’s guild is scarce and outdated. Recently, a guild-based protocol to target spiders as potential natural enemies of *P. spumarius* was developed by Benhadi-Marín et al.^15^. The protocol focused on finding dominant guilds of spiders in olive groves and analyzing their functional response towards *P. spumarius*. However, not always prey choice by predators in field conditions can sometimes be established by using direct observation. Thus, to achieve a more accurate identification of *P. spumarius* natural enemies, a gut analytical method enabling field assessment of predation is required.

Polymerase chain reaction (PCR)-based techniques are valuable tools in ecological studies, namely in studying interactions between the pest and its natural enemies^16–19^. By using taxon-specific primers, PCR-based techniques allow detecting specific ingested preys in the diet of predators since their DNA remains in the predator's gut before totally digested^20^. However, the effectiveness and sensitivity of such approach should take into account additional issues. The choice of target markers and the length of the sequence region to amplify are some aspects that need to be taken into account. Indeed, it is expected that degradation of the DNA of consumed preys will occur throughout the digestive process. In these conditions, approaches targeting cell-abundant, small multi-copy DNA fragments, like mitochondrial DNA, are preferable^21^. Another methodological aspect to be considered on PCR-based analysis of predation includes the type of predator tissue to be used to extract DNA^19^. The predator's digestive tract is the preferred source to extract DNA from ingested preys^18,22–24^. However, gut dissection in spiders is not possible. They have branching digestive tracts into highly complex diverticulum extending throughout the whole body, including their legs; therefore, digestion takes place in different parts of the body^25^. Nevertheless, when performing molecular gut-content analyses in spiders, the extraction of the whole body or just the abdomen, which has a higher proportion of prey DNA, is necessary^26^. In this approach, the underrepresented and degraded DNA from the prey can be masked by the overabundant DNA of the predator^27^. Aside from these issues, prey DNA digestion rates and the species of the predator^28,29^ can influence post-feeding prey detection periods in predators’ guts.

The main goal of this work was to design and evaluate taxon-specific primers targeting the mitochondrial cytochrome oxidase I (COI) and cytochrome b (*cyt*B) genes, to be used for a PCR-based diagnostic method, to detect *P. spumarius* within spiders. Feeding experiments were performed to evaluate the effectiveness of this DNA-based diagnostic tool. Specifically, this work analysed: (1) the suitability of the molecular marker selected regions and the specificity and sensitivity of the designed primers on *P. spumarius* detection; (2) the prey detectability over time in the spider *Xysticus acerbus* Thorell, 1872 (Thomisidae) using DNA extracts from their body; (3) and the efficiency of the designed primers to detect *Philaenus* in *Oxyopes* sp. (Oxyopidae) spiders directly collected from the field.

## Results

### Primer specificity and sensitivity

**Figure 1:**
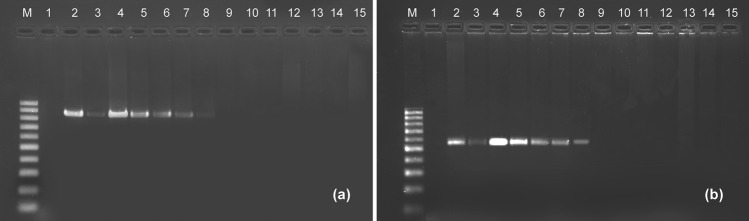
Primer’s specificity and sensitivity for *Philaenus*. Agarose gel electrophoresis of amplification products with primer pairs (**a**) COI_Ph71F/COI_Ph941R and (**b**) cytB_Ph85F/cytB_Ph635R. Lane M: Molecular Weight Marker 100 bp DNA Ladder (BIORON, GmbH); Lane 1: Mock community without DNA of *P. spumarius* added; lane 2: Mock community with 10 ng/μL DNA of *P. spumarius*; lane 3: Mock community with 0.1 ng/μL DNA of *P. spumarius*; lane 4: *P. spumarius* with DNA at the extracted concentration; lane 5 to 7: different specimens of *P. spumarius* at 10 ng/μL; lane 8: *P. spumarius* at 0.1 ng/μL; lane 9: *N. campestris* (10 ng/μL); lane 10: *N. lineatus* (10 ng/μL); lane 11: *Aphrophora* sp. (10 ng/μL); lane 12: *L. coleoptrata* (10 ng/μL); lane 13: *C. viridis* (10 ng/μL); lane 14: *Cercopis* sp. (10 ng/uL); lane 15: *X. acerbus* (10 ng/μL).

The specificity of the 20 primer pairs chiefly designed for *Philaenus spumarius* (Supplementary TableS1) was first tested. In total, 19 primer pairs successfully yielded DNA fragments of the expected size, although a few amplicons showed faint and/or double bands (Supplementary Table S2). Seven primer pairs gave clear single bands and high annealing temperatures and were further tested for specificity and sensitivity. Among them, the four primer pairs COI_Ph71F/COI_Ph937R, COI_Ph71F/COI_Ph941R, cytB_Ph85F/cytB_Ph551R and cytB_Ph85F/cytB_Ph635R showed to be highly specific for *P. spumarius*, without non-specific amplifications (Supplementary Table S3). Experiments with diluted DNA concentrations from *P. spumarius* and spiked mock samples indicated that the primer pairs COI_Ph71F/COI_Ph941R and cytB_Ph85F/cytB_Ph635R, were the most sensitive and efficient (Supplementary Table S3). Both primer sets showed the capacity to amplify *P. spumarius* DNA at low concentrations of 0.1 ng/μL, including when mixed with large quantities of non-target species DNA (Fig. 1).

Sequencing of the amplified fragments confirmed the specificity of amplification, showing 99% identity with *P. spumarius* sequences in GenBank. COI_Ph71F/COI_Ph941R set of primer generates a PCR product of 870 bp (Fig. 1), and its high specificity and sensitivity was achieved with the following PCR cycling conditions: an initial denaturation for 3 min at 94 °C, followed by 30 cycles at 94 °C for 30 s, 64 °C for 30 s, 72 °C for 40 s and a final extension at 72 °C for 7 min. The primer pair cytB_Ph85F/cytB_Ph635R generates an amplicon with 550 bp (Fig. 1). Optimized amplification conditions were: initial denaturation step for 3 min at 94 °C, followed by 30 cycles at 94 °C for 40 s, 64 °C for 40 s, 72 °C for 30 s, and a final extension at 72 °C for 7 min. Thus, the suitability of these two pairs of primers to detect *P. spumarius* DNA in feeding assays was further evaluated.

### Feeding assay and digestion of *P. spumarius*

**Figure 2:**
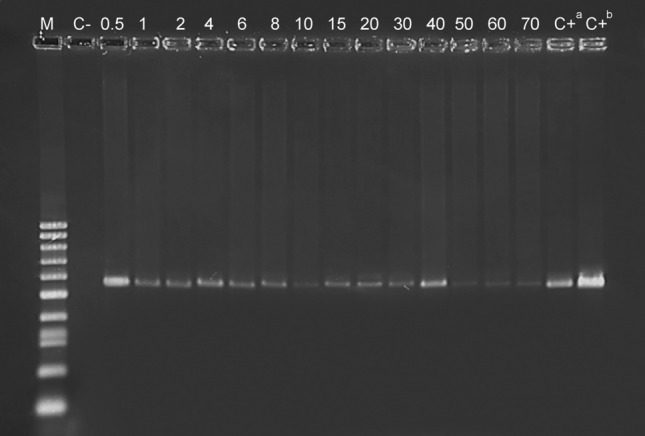
PCR amplification products obtained from DNA samples extracted in different post-feeding times by using the primer pair cytB_Ph85F/cytB_Ph635R (expected lenght: 550 bp). Lane M: Molecular weight marker: 100 bp DNA Ladder (BIORON, GmbH); Lane C-: *X. acerbus*; Lanes 0.50, 1, 2, 4, 6, 8, 10, 15, 20, 30, 40, 50, 60 and 70 are the post-feeding times; Lane C + a mix of DNA of *X. acerbus* and *P. spumarius*, in a proportion 3:1; Lane C + b: *P. spumarius*.

**Figure 3:**
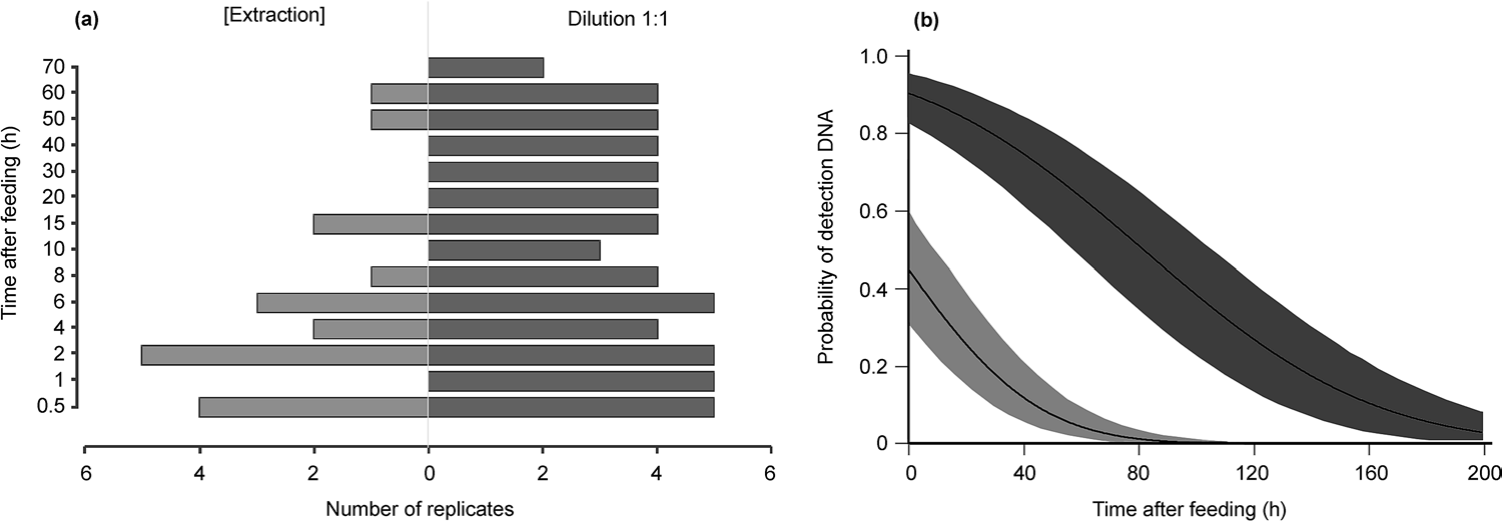
(**a**) Number of positive amplifications for detecting *P. spumarius* in the predator *X. acerbus* by comparing the use of gDNA at the extracted concentration (light gray bars) and gDNA diluted in 1:1 proportion (dark gray bars) at different post-feeding times (0.3–70 h). PCRs were performed using primer pair: cytB_Ph85F/cytB_Ph635R. (**b**) *P. spumarius* DNA detection probability curves in *X. acerbus* specimens after feeding. Lines are fitted Probit model, and the bands surrounding them represent the limits of the 95% confidence interval of the curves. Light gray curve: probability of obtaining positive amplifications when no DNA dilution is done; dark gray curve: probability of obtaining positive amplifications when the DNA is diluted in a 1:1 ratio.

The COI_Ph71F/COI_Ph941R and cytB_Ph85F/cytB_Ph635R primer pairs were used in the feeding assays to detect the presence of *P. spumarius* in the gut of *X. acerbus* specimens. The cytB_Ph85F/cytB_Ph635R primer pair proved to be the most efficient in detecting the presence of *P. spumarius* (Fig. 2), since no positive amplifications were observed when the COI_Ph71F/COI_Ph941R primer pair was used in the feeding assays (data not shown). However, the detection of *P. spumarius* DNA with cytB_Ph85F/cytB_Ph635R primer pair was higher with DNA dilution (1:1) than when used at the extracted concentration (Fig. 3a). The detection of *P. spumarius* DNA following consumption by *X. acerbus* significantly decreased with the post-feeding time (X^2^ = 9.806, df = 1, *p* = 0.0017 when no DNA dilution is done; and X^2^ = 4.59, df = 1, *p* = 0.0321 when a DNA dilution, in a proportion 1:1, is made) (Fig. 3b). According to Probit regression, in the diluted DNA samples, the *P. spumarius* DNA could be detected in 85% of cases within up to 20 h of digestion, decreasing to 50% after 82 h (Fig. 3b).

### Field assay

A total of 50 *Oxyopes* sp. individuals were analysed to confirm the possible predation of *Philaenus* in the field. This analysis was performed using the primer pair cytB_Ph85F/cytB_Ph635R. Twenty percent of the spiders tested positive. Sequence analysis of amplified products confirmed that all the positive spiders ingested *P. spumarius*.

### Phylogenetic analyses

COI and *cyt*B sequences from this study and all the GenBank-retrieved sequences used in the multiple-alignment for the primers design were subjected to phylogenetic analyses. The purpose of these analyses was to expose the relationship among the organisms and to compare the phylogenetic resolution of each DNA molecular marker. The COI sequences of the species specimens used in this study confirmed the initial identification based on morphological traits. Molecular analyses confirmed that used species effectively belonged to different clades and were correctly identified (Supplementary Fig. 2A). The three *P. spumarius* sequences studied cluster in a subclade, within a large clade encompassing all the *Philaenus* spp. sequences included in the alignment. The same pattern was observed for the *cyt*B phylogeny (Supplementary Fig. 2B), with all the *P. spumarius* sequences from this study also grouped in a subclade. This molecular marker shows a higher level of genetic divergence of the *Philaenus* clade in relation to its relatives when compared to COI.

## Discussion

In this study, we successfully developed a PCR-based diagnostic assay for *Philaenus spumarius,* although its species-specific capability could not be tested. Among twenty primer sets tested, the primer pairs COI_Ph71F/COI_Ph941R and cytB_Ph85F/cytB_Ph635R showed sensitivity and specificity to DNA from *P. spumarius*. Both primers sets detected *P. spumarius* in a mixture of DNA from different organisms, at concentrations 100-fold lower than non-target DNA species (including species belonging to the same family of *Philaenus*). These primer pairs target regions from two mitochondrial protein-coding genes, *i.e.* the standard barcode for invertebrates, cytochrome c oxidase subunit I (COI_Ph71F/COI_Ph941R), and the cytochrome b (cytB_Ph85F/cytB_Ph635R) genes. Currently, the COI gene is one of the most used markers for PCR-based gut-content analysis in arthropods^17,18,23,30–32^, including in *Philaenus* detection^24^. On the contrary, the *cyt*B has rarely been used for species identification of invertebrates but is widely used within vertebrates^16^. In fact, no previous work has explored this gene for PCR-based detection and identification of prey consumed by arthropod predators. However, our results indicated that *cyt*B has a great potential for this type of diagnostic, even showing a higher discriminatory power to distinguish the *Philaenus* clade than COI (Supplementary Fig. S2). More importantly, among the two primer pairs selected, only cytB_Ph85F/cytB_Ph635R detected the presence of *P. spumarius* on the feeding assays. This result may be related to differences in the lenght of the sequence amplified by the two primer pairs and differential DNA digestion, as observed in marine invertebrates^33^. Overall, our results suggested that the length of the sequence amplified is crucial to successfully detect *P. spumarius* DNA in the gut of *X. acerbus*, as previously reported for other prey-predator combinations^22,34–36^. It is likely that the prey DNA molecules are broken into smaller fragments during digestion, as also previously reported in other spider diet analyses^32,37^. Thus, the cytB_Ph85F/cytB_Ph635R primer pair that amplifies a shorter fragment (550 bp) would likely improve detection success when compared to primer pair COI_Ph71F/COI_Ph941R that amplify longer fragments (870 pb); even if longer fragments (up to 600 bp) are readily amplified from predators’ guts^17,34,35^.

Prey detectability is, in most studies, focused on small-sized spiders by homogenizing the whole body or just the abdomen to reduce the predator DNA^17,23,38–40^. Here, the digestion activity of *X. acerbus* specimens was successfully followed by applying the conceived diagnostic PCR assay in the DNA extracted from the entire body of the predator. However, the detection of *P. spumarius* from whole-body DNA extracts of *X. acerbus* showed to be greater in DNA samples diluted 1:1 compared with non-diluted ones. Therefore, we hypothesized that this phenomenon is due to the reduction of PCR inhibitors, namely polysaccharides and proteins (which are main constituents of arthropods)^41^, through DNA dilution. Furthermore, this procedure may also reduce the amount of non-target DNA of the predator, which at high concentrations can inhibit prey detection^22,32^. Therefore, dilution of DNA to reduce the predator's DNA concentration seems to be an important procedure to enhance PCR amplification.

The present study showed that digestion time is an important aspect of the detectability of prey DNA, consistent with previous studies for other groups of predators and prey^28,35,37^. Understanding how quickly the level of prey DNA decreases inside a predator and identifying the digestion time where there is a 50% of detection success are essential to analyse predators sampled in the field^37^. This information can be used together with knowledge of predator activity to plan the best hour in the day to collect them in the field. Spiders are generally nocturnal^42^, so it is expected that specimens collected early in the day may lead to high prey detection rates. Accordingly, the DNA in their guts is not digested since feeding the night before. Our diagnostic PCR assay allows the detection of *P. spumarius* in *X. acerbus* at least up to 70 h post-feeding, and based on probit model, it can reach 50% detection success after 82 h. The capacity of spiders to store excess food in the branching of the midgut for extended periods^43^ can probably justify the long detection time observed. Sint et al.^17^, Monzó et al*.*^23^, and Hosseini et al.^37^ also reported long detection periods in other spider species (49.6, 78.25, and 79.2 h, respectively). Considering that spiders are efficient predators and have higher average detection times when compared to other arthropods makes them prone to gut content analyses to accurately detect prey in specimens collected in the field. Indeed, this was corroborated by our field assay, which successfully confirmed the predation of *Philaenus* by the spider *Oxyopes* sp. naturally occurring in its ecosystem. Lantero et al.^24^ have previously designed specific primers for *P. spumarius* based on the COI sequence region. In their work, no feeding assay was developed to establish the prey detectability over time, and the DNA was extract from the gut. By taking advantage of complete mitogenomes now available for Aphrophoridae species, we could explore others COI sequence regions (see Supplementary Fig. S1) that could be suitable to design novel primers. Also, COI primers designed in our study were developed to amplify a larger fragment than those from Lantero et al*.*^24^, since an unequivocal identification of *Philaenus* at the species level may not be feasible by using small COI fragments (Seabra et al.^44^; see also Supplementary Fig. S2). This is relevant for gut DNA derived from field samples whenever a confirmation by sequencing is required. Indeed, as in Lantero et al*.*^24^ we did not evaluate the primers on other *Philaenus* species. So, we cannot assure that the primers from this study are species-specific for *P. spumarius*, although the Primer-BLAST results indicated a good likelihood for it. Another advantage of our primers, and notably that of the primer pair cytB_Ph85F/cytB_Ph635R, is related to the length of their PCR products. This primer amplifies a 550 bp DNA fragment *P. spumarius* from the digestive tract of the two studied spider species, making it feasible to be sequenced for molecular/phylogenetic analysis, thus allowing further confirmation of the identification.

In conclusion, the *Philaenus* primers designed and the optimized PCR-based diagnostic assay can provide an effective and sensitive method for detecting potential predators of the main vector (or its very close phylogenetic relatives) of *X. fastidiosa*. This PCR-based diagnostic assay can help implement more sustainable measures to limit the spread of this vector-borne pathogen. We also reinforce the importance of spiders as predators, and particularly as a natural enemy of *Philaenus* in the field.

## Materials and methods

### Collection and molecular identification of the arthropods

In order to evaluate the ability of the designed primer pairs to specifically amplify DNA from *Philaenus*, adults of *P. spumarius* were collected to act as a positive control. *Neophilaenus campestris* (Fallen, 1805) (Aphrophoridae), *Neophilaenus lineatus* (Linnaeus, 1758) (Aphrophoridae), *Lepyronia coleoptrata* (Linnaeus, 1758) (Aphrophoridae), *Aphrophora* sp. (Aphrophoridae), *Cicadella viridis* (Linnaeus, 1758) (Cicadellidae), *Cercopis* sp. (Cercopidae), and the spider *Xysticus acerbus* were also collected to be used as non-target species. Arthropod’s collection was carried out in the natural ground vegetation with an entomological sweep net (38 cm diameter), in the *Campus* of Instituto Politécnico de Bragança (41° 47′ 53.2″ N, 6° 45′ 51.5″ W), between April and July of 2019. The arthropods were initially identified to the genus/species level using a binocular stereoscopic, preserved in absolute ethanol, and stored at − 20 °C, until subsequent DNA extraction. To confirm the identification of the arthropods, a molecular-based approach was followed. All the insects were homogenised in liquid nitrogen, and the genomic DNA was extracted using the SpeedTools tissue DNA extraction kit (Biotools, Spain) following the manufacturer's instructions. The barcode region of the mitochondrial COI gene was amplified using the universal primers LCO1490/HCO2198^45^. Amplifications were run in a MyCycler™ Thermocycler (Bio-Rad) using 20 μL PCR reactions, which contained 1 × buffer, 2.5 mM of MgCl_2_, 200 µM of dNTPs, 0.2 µM of each primer, and 1.25 U of Taq DNA polymerase (BIORON, GmbH). Cycling conditions were: initial denaturation at 94 °C for 5 min, followed by 35 cycles of 94 °C for 45 s, 50 °C for 1 min and 72 °C for 1 min, with a final extension of 72 °C for 10 min. PCR products (~ 710 bp) were run on a 1% (v/v) agarose gel stained with 1X Gel Red™ nucleic acid gel stain (Biotium, California, USA), and the amplified products were purified and sequenced at Macrogen Inc. (Madrid, Spain). The DNA sequences were analysed and edited with MEGA v10.1.8^46^, and the identification of each specimen was confirmed by querying the GenBank database using the Nucleotide Basic Local Alignment Search Tool (BLASTn) in NCBI’s website (www.ncbi.nlm.nih.gov).

### Design of *Philaenus*-specific primers and development of diagnostic PCRs

**Figure 4:**
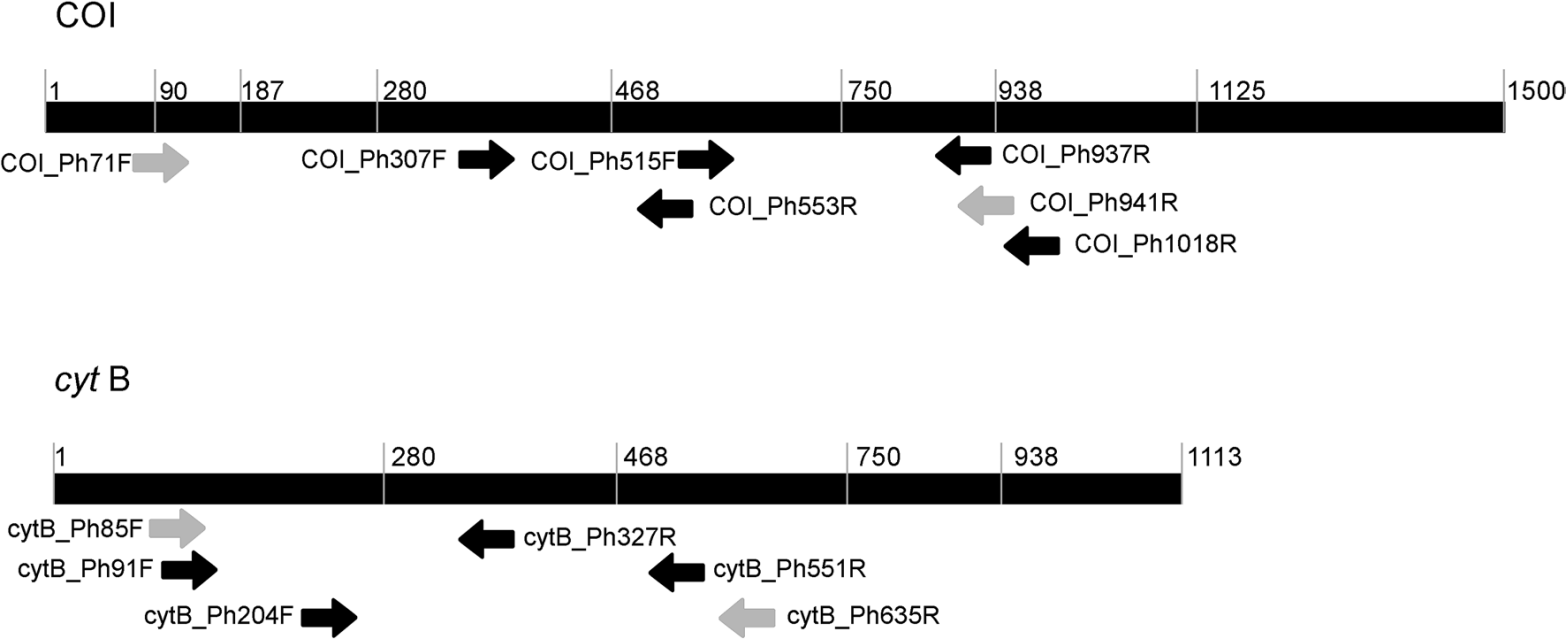
Schematic diagram of COI and *cyt*B gene regions showing the binding sites of the PCR primers developed and tested in this study. Primers were designed on conserved regions of *P. spumarius* sequences. All possible combinations were tested (see also Supplementary Table S1 for additional details, e.g. amplicon expected lenght). Nucleotide positions are according to full COI (NC_005944:1382-2915; 1534 nt) and *cyt*B (NC_005944:10,216-11,348; 1133 nt) gene sequences retrieved from the complete mitochondrial genome of *Philaenus spumarius*. White arrows represent primers selected for further experiments.

Publicly available full (extracted from mitochondrial genomes) or near-full sequences of COI and *cyt*B genes from *Philaenus* spp. and close-related taxa representatives (see Supplementary Fig. S1) were reached from GenBank. Due to the small number of sequences present in the database, partial COI sequences from *Philaenus* specimens covering either the 5’ or the 3’ part of the gene were additionally retrieved from GenBank (Supplementary Fig. S1). All sequences were then aligned in Geneious 8.1.8 (Biomatter, New Zealand) by using the algorithm ClustalW. Primer3 2.3.4.^47^, a tool available in Geneious, was then used for both datasets to identify potential gene regions suitable for the design of *Philaenus*-specific primers. Additional regions were also visually inspected for adequacy, being all candidate primers chosen or redesigned manually. Primers properties (e.g., length, melting temperature, GC content) were evaluated with Geneious and OligoEvaluator, a Sigma-Aldrich accessible tool (http://www.oligoevaluator.com) and analysed for secondary structures (including hairpins, self-dimers, and cross-dimers) formation in primer pairs with the online OligoAnalyzer™ tool (www.idtdna.com). The specificity of the primer pairs was virtually assessed with Primer-BLAST^48^. The shortlist of the best oligonucleotide candidates, 7 targeting the COI gene and 6 targeting *cyt*B gene (Table 1 and Fig. 4) were synthesized at Frilabo (Portugal), tested, and their PCR conditions optimised.

**Table 1:** Primers from COI and *cyt*B genes designed to amplify specifically *Philaenus* and tested in this study.

Primer names	Sequences (5′–3′)
COI_Ph71F	CTGGAATAATTGGGACTACTC
COI_Ph307F	CTTCCTCCTTCGTTAACGC
COI_Ph515F	CAGGTATGAAAATAGATCG
COI_Ph553R	CGATCTATTTTCATACCTG
COI_Ph937R	CAGCAATAATTATTGTGGC
COI_Ph941R	GGTACAGCAATAATTATTGTGG
COI_Ph1018R	AGGAGAAGACAATTTG
cytB_Ph85F	GTCATAGGAGTAATAATTATACTGACAG
cytB_Ph91F	GGAGTAATAATTATACTGACAG
cytB_Ph204F	TCCTTACCTCGGAGAATC
cytB_Ph327R	GCTTCTTATAACTAACAC
cytB_Ph551R	TTAATGTGGGCAGGGGTG
cytB_Ph635R	GATATGATTAATGCAATTACCCC

Evaluation of the primer sets' specificity and sensitivity and their efficacy as a diagnostic tool were performed for all possible primer pair combinations (Supplementary Table S1) in PCR assays. The specificity of the primers to *Philaenus* was evaluated by using as templates genomic DNA extracted from *P. spumarius* and from seven non-target species (including closely-related taxa of *Philaenus*). Primer sensitivity was assessed using different concentrations of *P. spumarius* DNA (*i.e*., at extraction concentration of 121.43 ng/μL, and diluted at 10 ng/μL and 0.1 ng/μL). For the evaluation of primers' efficiency three different sample types were prepared: (1) a mock sample with a mixture of DNA of the 7 non-target species at equal ratios and concentrations (10 ng/μL each); and mock samples spiked with DNA of *P. spumarius* at (2) 10 ng/μL; (3) and 0.1 ng/μL. For all PCR assays, each primer pair was used in 10 µL reactions, containing 1 × buffer, 2.5 mM of MgCl_2_, 1.5 mg/mL of bovine serum albumin (BSA, Promega), 200 µM of dNTP’s, 0.2 µM of each primer, and 1.25 U Taq DNA polymerase (BIORON, GmbH). The PCR program was optimized by varying the annealing temperature (from 48 to 64 °C through gradient PCR; Supplementary Table S2). Primer sets showing good performance at higher annealing temperatures were then subjected to further tests and optimizations to improve the specificity and sensitivity by varying the time of denaturation, annealing and extension (Supplementary Table S3). Optimized cycling protocols are indicated in the results section for the selected primer pairs.

### Post feeding detection period of *P. spumarius* in *X. acerbus*

Feeding trials were conducted to determine when *Philaenus* DNA is detectable within the spider *X. acerbus* after feeding. Accordingly, live adults of *X. acerbus* were collected in natural ground vegetation with an entomological sweep net (38 cm diameter) in a meadow located in Rabal (41° 51′ 30.8″ N, 6° 44′ 53.4″ W), near Bragança (Northeastern Portugal), in July of 2019. Spiders were brought to the laboratory, where they were identified to species level and individually placed in Petri dishes (7 cm diameter) to be starved for seven days. During this period, the spiders were maintained at 21 °C with 55% relative humidity and 16:8 h (L:D) photoperiod, and water was supplied daily. At the end of the starvation period, one *P. spumarius* adult, provided by the Spanish National Research Council (CSIC, Madrid), was offered to each spider. Predators were observed until feeding started and ceased (8.2 ± 0.22 h). At 0.5, 1, 2, 4, 6, 8, 10, 15, 20, 30, 40, 50, 60, and 70 h post-feeding, specimens of *X. acerbus* were sacrificed, stored in 96% ethanol, and frozen at − 20 °C until subsequent molecular assay. At each post-feeding time, five replicates were conducted and processed independently. After being macerated in liquid nitrogen, DNA from each spider was extracted using the SpeedTools DNA Tissue Extraction kit (Biotools, Spain), according to manufacturer instructions. By using the whole body of the spiders for extraction, DNA of *P. spumarius* may be sticking on the legs or abdomen, which can lead to subsequent false positives. Therefore, before maceration, spiders were externally cleaned several times with 96% ethanol and dried on filter paper. Gut content PCR amplification of the *P. spumarius* DNA from the feeding trial was performed by using the two primer pairs COI_Ph71F/COI_Ph941R and cytB_Ph85F/cytB_Ph635R, and the respective optimized PCR condition, which is described in the results section. PCR reactions were performed with DNA at the extraction concentration (240.81 ± 117.80 ng/μL) and diluted in a proportion of 1:1. In each PCR assay, DNA extracted from *P. spumarius* specimens and a mix of *X. acerbus* and *P. spumarius* DNA, in a proportion 3:1, was used as a positive control (C +). DNA extracted from *X. acerbus* starved for seven days was used as negative control (C −). PCR products were separated and visualized as previously described. Positive scores of PCR amplification were subjected to Probit analysis using MedCalc statistical software version 19.4.1 to calculate the time limit of *P. spumarius* detectability after consumption by *X. acerbus*. Chi-square (X^2^) tests were performed to determine data fitting to the Probit model.

### Field assay

The applicability of the PCR-based technique developed in this work was tested by screening 50 *Oxyopes* sp. spiders. A different spider was selected to corroborate the specificity of the primers. *Oxyopes* sp. spiders were collected in an olive grove under integrated production management located in Mirandela region (Northeastern Portugal) (41° 29′ 15.77″ N, 7° 07′ 52.11″ W), in mid-July 2019. This sampling grove was selected due to the previously reported presence of *P. spumarius*^49^. Adults of *Oxyopes* sp. were collected on ground cover vegetation with an entomological sweep net (38 cm diameter) and individually selected with a mouth aspirator. All collected spiders were immediately placed in falcon tubes with 96% ethanol, morphologically identified, and frozen at − 20 °C for later DNA extraction. Total DNA was extracted from the whole spiders using the SpeedTools DNA tissue extraction kit (Biotools), following the manufacturer’s guidelines. DNA diluted in a proportion of 1:1 from each spider was amplified by using the cytB_Ph85F/cytB_Ph635R primer pair and its optimized PCR conditions, to confirm the possible predation of *Philaenus* sp. in the field. Each reaction was checked by electrophoresis, and the positive samples were sequenced at Macrogen Inc. (Madrid, Spain) for taxonomic molecular-based identification, following the same procedure as mentioned above.

### Phylogenetic relationship among specimens

COI and *cyt*B sequences from this study and all the GenBank-retrieved sequences used in the multiple-alignment for the primers design were aligned using ClustalW, in MEGA v10.1.8^45^. A Neighbor-Joining (NJ) tree with 5000 bootstrap replicates was constructed to each multiple-alignment, using the same software. Phylogenetic trees were edited with Inkscape 0.92 (www.inkscape.org).

## Supplementary Information

Supplementary Information.
