# Supplementary material for: A novel molecular diagnostic method for the gut content analysis of Philaenus DNA
Source: Sci Rep. 2022 Jan 11;12:492. doi: 10.1038/s41598-021-04422-1 (PMC8752687; doi:10.1038/s41598-021-04422-1)
Supplement: Supplementary file 1 — Supplementary Information. [file 41598_2021_4422_MOESM1_ESM.docx]

**Supplementary Material**

**A novel molecular diagnostic method for gut content analysis of *Philaenus* DNA**

Isabel Rodrigues^a,b^, Vítor Ramos^a^, Jacinto Benhadi-Marín^a^, Aránzazu Moreno^c^, Alberto Fereres^c^ José Alberto Pereira^a^, Paula Baptista^a^*

^a^ Centro de Investigação de Montanha (CIMO), Instituto Politécnico de Bragança. *Campus* de Santa Apolónia, 5300-253 Bragança, Portugal.

^b^ Universidad de Léon, Departamento de Ingeniería Agrária, Av. Portugal, n° 41, 24071 Léon, Spain.

^c^Instituto de Ciencias Agrarias, Consejo Superior de Investigaciones Científicas (ICA-CSIC), C/Serrano 115 dpdo, 28006 Madrid, Spain

* Corresponding author

Paula Baptista

Tel.: + 351 273303332; Fax + 351 273 325405.

E-mail address: pbaptista@ipb.pt

## Supplementary figures

**COI**

***Cyt*B**

**Supplementary Figure S1.** Alignments of COI and *cyt*B gene sequences from different xylem feeding insects from the Aphrophoridae and Cercopidae families. Positions according to the COI gene or the mitogenome sequence (for cytB) of P. spumarius (NC_005944). Primers were designed on conserved regions for P. spumarius.

##
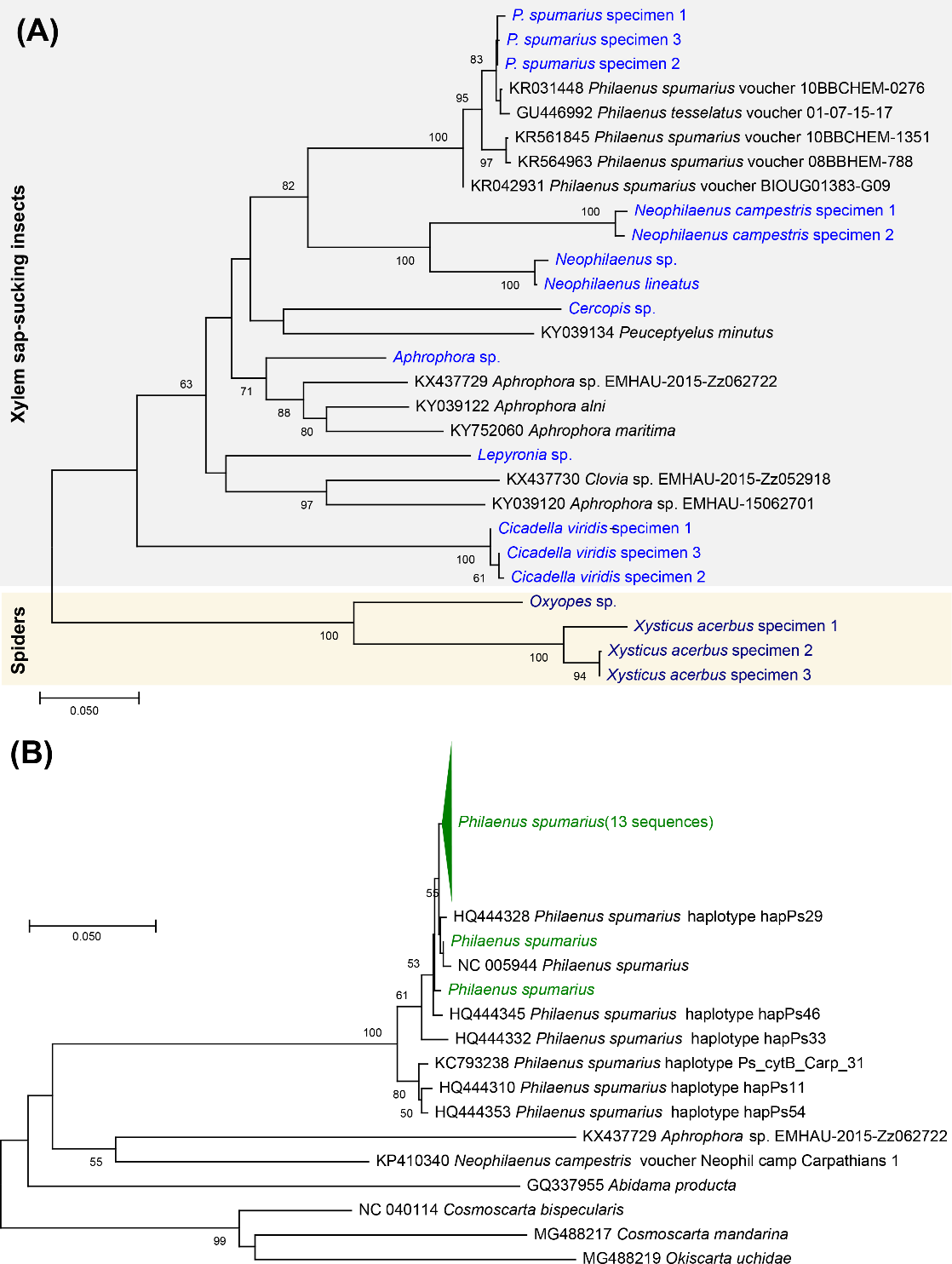


**Supplementary Figure S2.** NJ phylogenetic trees for species studied in this work (colored sequences) and their relation to species included in the primer designing (sequences in black, with accession numbers). (**A**) COI sequences PCR-amplified in this study with the universal primers from Folmer et al. (1994) are highlighted in blue. (**B**) *Cyt*B sequences obtained in this study (in green) were amplified with the newly developed primer pair cytB_Ph85F/cytB_Ph635R. There were a total of 681 (A) and 665 (B) positions in the final datasets. Bootstrap values (5000 replicates) are shown next to branch nodes. The scale bar indicates the number of base substitutions per site.

## Supplementary tables

**Supplementary Table S1.** Primer sets tested in this study and their estimated PCR product sizes. In bold are the selected primer pairs used in post feeding and field assays.

| **Forward** | **Reverse** | **Size (bp)** |
| --- | --- | --- |
| COI_Ph71F | COI_Ph553R | 482 |
| COI_Ph71F | COI_Ph937R | 866 |
| **COI_Ph71F** | **COI_Ph941R** | **870** |
| COI_Ph71F | COI_Ph1018R | 947 |
| COI_Ph307F | COI_Ph553R | 246 |
| COI_Ph307F | COI_Ph937R | 630 |
| COI_Ph307F | COI_Ph941R | 634 |
| COI_Ph307F | COI_Ph1018R | 711 |
| COI_Ph515F | COI_Ph937R | 422 |
| COI_Ph515F | COI_Ph941R | 426 |
| COI_Ph515F | COI_Ph1018R | 503 |
|  |  |  |
| cytB_Ph85F | cytB_Ph327R | 242 |
| cytB_Ph85F | cytB_Ph551R | 466 |
| **cytB_Ph85F** | **cytB_Ph635R** | **550** |
| cytB_Ph91F | cytB_Ph327R | 236 |
| cytB_Ph91F | cytB_Ph551R | 460 |
| cytB_Ph91F | cytB_Ph635R | 544 |
| cytB_Ph204F | cytB_Ph327R | 123 |
| cytB_Ph204F | cytB_Ph551R | 347 |
| cytB_Ph204F | cytB_Ph635R | 431 |

**Supplementary Table S2.** Different annealing temperatures tested for each primer set to improve the specificity to *Philaenus*.

| **Primer set** | **PCR condition A** | | | | | |
| --- | --- | --- | --- | --- | --- | --- |
|  | 48 ºC | 49.7 ºC | 52.2 ºC | 55.5 ºC | 59.9 ºC | 64 ºC |
| COI_Ph71F - COI_Ph553R | + | + | + | +^*^ | +^*^ | +^*^ |
| COI_Ph71F - COI_Ph937R | + | + | + | + | + | + |
| COI_Ph71F - COI_Ph941R | + | + | + | + | + | + |
| COI_Ph71F - COI_Ph1018R | + | + | + | +^$^ | - | - |
| COI_Ph307F - COI_Ph553R | +^$*^ | +^$*^ | +^$*^ | +^$*^ | +^$*^ | - |
| COI_Ph307F - COI_Ph937R | + | + | + | + | + | + |
| COI_Ph307F - COI_Ph941R | + | + | + | + | + | + |
| COI_Ph307F - COI_Ph1018R | +^$*^ | +^$*^ | +^$*^ | +^$*^ | - | - |
| COI_Ph515F - COI_Ph937R | + | + | + | + | +^$*^ | - |
| COI_Ph515F - COI_Ph941R | + | + | + | +^$^ | +^$*^ | - |
| COI_Ph515F - COI_Ph1018R | + | + | + | +^$^ | - | - |
| cytB_Ph85F - cytB_Ph327R | +^$*^ | +^$*^ | +^$*^ | +^$*^ | +^$^ | - |
| cytB_Ph85F - cytB_Ph551R | + | + | + | + | + | + |
| cytB_Ph85F - cytB_Ph635R | + | + | + | + | + | + |
| cytB_Ph91F - cytB_Ph327R | + | +^*^ | +^*^ | +^*^ | - | - |
| cytB_Ph91F - cytB_Ph551R | + | + | +^$^ | +^$^ | - | - |
| cytB_Ph91F - cytB_Ph635R | - | - | - | - | - | - |
| cytB_Ph204F - cytB_Ph327R | +^*^ | +^*^ | +^*^ | +^*^ | - | - |
| cytB_Ph204F - cytB_Ph551R | +^*^ | +^*^ | +^*^ | +^*^ | +^*^ | +^*^ |
| cytB_Ph204F - cytB_Ph635R | +^$^ | +^$^ | +^$^ | +^$^ | +^$^ | - |

**PCR condition A:** initial denaturation for 3 min at 94°C, followed by 30 cycles at 94°C for 40 s, 48 to 64°C for 40 s, 72°C for 45 s and a final extension at 72°C for 7 min.

**+:** positive amplification; **- :** negative amplification; **$:** faint band;*: double band.

: amplified products confirmed by sequencing.

|  | **PCR condition B** | | | | | | |  | **PCR condition C** | | | | | | | | | | | | | |  | | **PCR condition D** | | | | | | | | | | | | | |
| --- | --- | --- | --- | --- | --- | --- | --- | --- | --- | --- | --- | --- | --- | --- | --- | --- | --- | --- | --- | --- | --- | --- | --- | --- | --- | --- | --- | --- | --- | --- | --- | --- | --- | --- | --- | --- | --- | --- |
| Primer set gDNA | P. sp^Ext^ | P. sp^10^ | P. sp^0.1^ | N.ca^10^ | Mock  1 | Mock  2 | Mock  3 |  | P. sp^Ext^ | P. sp^10^ | P. sp^0.1^ | N.ca^10^ | N. li^10^ | Aph^10^ | L. co^10^ | C. vi^10^ | Cer^10^ | X. ac^10^ | Mock  1 | Mock  2 | Mock  3 |  | | P. sp^Ext^ | | P. sp^10^ | P. sp^0.1^ | N.ca^10^ | N. li^10^ | Aph^10^ | L. co^10^ | C. vi^10^ | Ce^10^ | X. ac^10^ | Mock  1 | Mock  2 | Mock  3 |  |
| COI_Ph71F - COI_Ph937R | + | +^$^ | +^$^ | - | +^$^ | + | +^$^ |  | + | + (3/3)^$^ | - | - | - | - | - | - | - | - | - | + | +^$^ |  | | n.t. | | n.t. | n.t. | n.t. | n.t. | n.t. | n.t. | n.t. | n.t. | n.t. | n.t. | n.t. | n.t. |  |
| COI_Ph71F - COI_Ph941R | + | + | +^$^ | - | +^$^ | + | + |  | + | + (3/3) | +^$^ | - | - | - | - | - | - | - | - | + | + |  | | n.t. | | n.t. | n.t. | n.t. | n.t. | n.t. | n.t. | n.t. | n.t. | n.t. | n.t. | n.t. | n.t. |  |
| COI_Ph307F - COI_Ph937R | - | - | - | - | - | - | - |  | n.t. | n.t. | n.t. | n.t. | n.t. | n.t. | n.t. | n.t. | n.t. | n.t. | n.t. | n.t. | n.t. |  | | n.t. | | n.t. | n.t. | n.t. | n.t. | n.t. | n.t. | n.t. | n.t. | n.t. | n.t. | n.t. | n.t. |  |
| COI_Ph307F - COI_Ph941R | + | +^$^ | - | - | +^$^ | + | +^$^ |  | n.t. | n.t. | n.t. | n.t. | n.t. | n.t. | n.t. | n.t. | n.t. | n.t. | n.t. | n.t. | n.t. |  | | n.t. | | n.t. | n.t. | n.t. | n.t. | n.t. | n.t. | n.t. | n.t. | n.t. | n.t. | n.t. | n.t. |  |
| cytB_Ph85F - cytB_Ph551R | + | - | - | - | - | + | + |  | - | - | - | - | - | - | - | - | - | - | - | - | - |  | | +^$^ | | + (3/3)^$^ | - | - | - | - | - | - | - | - | - | - | - |  |
| cytB_Ph85F - cytB_Ph635R | + | + | +^$^ | - | +^$^ | + | + |  | - | - | - | - | - | - | - | - | - | - | - | - | - |  | | + | | + (3/3) | +^$^ | - | - | - | - | - | - | - | - | + | +^$^ |  |
| cytB_Ph204F - cytB_Ph635R | - | - | - | - | - | - | - |  | n.t. | n.t. | n.t. | n.t. | n.t. | n.t. | n.t. | n.t. | n.t. | n.t. | n.t. | n.t. | n.t. |  | | n.t. | | n.t. | n.t. | n.t. | n.t. | n.t. | n.t. | n.t. | n.t. | n.t. | n.t. | n.t. | n.t. |  |

**Supplementary Table S3.** PCR condition optimization for each primer set and DNA template. Primer sets showing good performance at higher annealing temperatures were subjected to further tests and optimizations to improve the specificity and sensitivity to *Philaenus*.

**PCR condition B:** initial denaturation for 3 min at 94°C, followed by 30 cycles at 94°C for 40 s, 64°C for 40 s, 72°C for 45 s and a final extension at 72°C for 7 min; **PCR condition C:** initial denaturation for 3 min at 94°C, followed by 30 cycles at 94°C for 30 s, 64°C for 30 s, 72°C for 40 s and a final extension at 72°C for 7 min; **PCR condition D:** initial denaturation for 3 min at 94°C, followed by 30 cycles at 94°C for 40 s, 64°C for 40 s, 72°C for 30 s and a final extension at 72°C for 7 min.

**P.sp**: *Philaenus spumarius;* **N.ca:** *Neophilaenus campestris;* **N.li**: *Neophilaenus lineatus*; **Aph:** *Aphrophora* sp.; **L.co:**^:^ *Lepyronia coleoptrata*; **C.vi**: *Cicadella viridis*; **Cer**: *Cercopis* sp.; **X.ac**: *Xysticus acerbus*. **Mock 1:** mix with gDNA of the non-target species in the same ratio; **Mock 2:** mix with gDNA of the non-target species in the same ratio and gDNA of *P. spumarius* at 10 ng/μL; **Mock 3:** mix with gDNA of the non-target species in the same ratio and gDNA of *P. spumarius* at 0.1 ng/μL.

**Ext**: [gDNA] at the extracted concentration (121.43 ng/μL.); **10**: [gDNA] at 10 ng/μL; **0.1**: [gDNA] at the 0.1 ng/μL.

**+:** positive amplification; **- :** negative amplification; **$:** faint band; **n.t.** not tested

In parentheses number of positive *vs* tested specimens.

: amplified products confirmed by sequencing.

**Supplementary Table S4.** Nucleotide Basic Local Alignment Search Tool (BLASTn) best-hit results for the different COI sequences (obtained with the universal primers) from specimens studied in this work.

| **Scientific name** | **Score** | **Query cover** | **E-value** | **Identity (%)** | **Accession** |
| --- | --- | --- | --- | --- | --- |
| *Cicadella viridis* | 1155 | 99% | 0.0 | 99.68% | [FR775764.1](https://www.ncbi.nlm.nih.gov/nucleotide/FR775764.1?report=genbank&log$=nucltop&blast_rank=1&RID=F9PJW5Z3016) |
| *Lepyronia coleoptrata* | 1062 | 100% | 0.0 | 98.35% | KF919404.1 |
| *Neophilaenus campestris* | 856 | 98% | 0.0 | 97.25% | [MK188544.1](https://www.ncbi.nlm.nih.gov/nucleotide/MK188544.1?report=genbank&log$=nucltop&blast_rank=1&RID=EENAS79D014) |
| *Neophilaenus lineatus* | 1046 | 99% | 0.0 | 97.09% | [KF920371.1](https://www.ncbi.nlm.nih.gov/nucleotide/KF920371.1?report=genbank&log$=nucltop&blast_rank=1&RID=F9NPRAUN01R) |
| *Oxyopes* sp. | 1027 | 94% | 0.0 | 98.62% | [MK644596.1](https://www.ncbi.nlm.nih.gov/nucleotide/MK644596.1?report=genbank&log$=nucltop&blast_rank=1&RID=EEPMSR4M014) |
| *Philaenus spumarius* | 1140 | 99% | 0.0 | 99.68 | [NC_005944.1](https://www.ncbi.nlm.nih.gov/nucleotide/NC_005944.1?report=genbank&log$=nucltop&blast_rank=1&RID=F9N02FNT016) |
| *Philaenus spumarius* | 822 | 100% | 0.0 | 99.56% | [HQ444308.1](https://www.ncbi.nlm.nih.gov/nucleotide/HQ444308.1?report=genbank&log$=nucltop&blast_rank=1&RID=MU4ET3G5016) |
| *Philaenus spumarius** | 383 | 93% | 4.00E-10^2^ | 88.46% | [MG406615.1](https://www.ncbi.nlm.nih.gov/nucleotide/MG406615.1?report=genbank&log$=nucltop&blast_rank=1&RID=H3CWBGN1016) |
| *Xysticus acerbus* | 983 | 98% | 0.0 | 95.60% | [KY268806.1](https://www.ncbi.nlm.nih.gov/nucleotide/KY268806.1?report=genbank&log$=nucltop&blast_rank=1&RID=F9S532G7016) |

* bad quality sequence

**References**

Folmer, O., Black, M., Hoeh, W., Lutz, R. & Vrijenhoek, R. DNA primers for amplification of mitochondrial cytochrome c oxidase subunit I from diverse metazoan invertebrates. *Mol. Mar. Biol. Biotechnol*. **3**, 294–299 (1994).
